# Influence of the broadly neutralizing antibody VRC01 on HIV breakthrough virus populations in antibody-mediated prevention trials

**DOI:** 10.1038/s41467-026-70888-0

**Published:** 2026-04-03

**Authors:** Carolyn Williamson, Chivonne Moodley, Craig A. Magaret, Elena E. Giorgi, Morgane Rolland, Dylan H. Westfall, Anna Yssel, Wenjie Deng, Raabya Rossenkhan, Nonhlanhla N. Mkhize, Lennie Chen, Hong Zhao, Tanmoy Bhattacharya, Alec Pankow, Ben Murrell, Talita York, Asanda Gwashu-Nyangiwe, Nonkululeko Ndabambi, Ruwayhida Thebus, Paula Cohen, Bronwen Lambson, Haajira Kaldine, Sinethemba Bhebhe, Michal Juraska, Hongjun Bai, Allan C. deCamp, Maurine D. Miner, James Ludwig, Cindy Molitor, Nicolas Beaume, David Matten, Yunda Huang, Lily Zhang, Daniel B. Reeves, Bryan Mayer, Shelly T. Karuna, John A. Hural, Lynn Morris, David Montefiori, Roger E. Bumgarner, Penny L. Moore, Paul T. Edlefsen, Srilatha Edupuganti, Nyaradzo Mgodi, M. Juliana McElrath, Myron S. Cohen, Lawrence Corey, Peter B. Gilbert, James I. Mullins

**Affiliations:** 1 Institute of Infectious Disease and Molecular Medicine, Department of Pathology, Faculty of Health Sciences, University of Cape Town, Cape Town, South Africa; 2 Centre for the AIDS Programme of Research in South Africa (CAPRISA), University of KwaZulu Natal, Durban, South Africa; 3 National Health Laboratory Service, Cape Town, South Africa; 4 Vaccine and Infectious Disease Division, Fred Hutchinson Cancer Center, Seattle, WA USA; 5 U.S. Military HIV Research Program, Walter Reed Army Institute of Research, Silver Spring, MD USA; 6 Henry M. Jackson Foundation for the Advancement of Military Medicine, Bethesda, MD USA; 7 Department of Microbiology, University of Washington, Seattle, WA USA; 8 National Institute for Communicable Diseases of the National Health Laboratory Service, Johannesburg, South Africa; 9 SAMRC Antibody Immunity Research Unit, Faculty of Health Sciences, University of the Witwatersrand, Johannesburg, South Africa; 10 Theoretical Division, Los Alamos National Laboratory, Los Alamos, NM USA; 11 Department of Microbiology, Tumor, and Cell Biology, Karolinska Institutet, Solna, Sweden; 12 Department of Surgery, Duke University Medical Center, Durham, NC USA; 13 Department of Biostatistics, University of Washington, Seattle, WA USA; 14 Emory University, Atlanta, GA USA; 15 University of Zimbabwe College of Health Sciences Clinical Trials Research Centre, Harare, Zimbabwe; 16 Department of Medicine, University of North Carolina at Chapel Hill, Chapel Hill, NC USA; 17 Department of Medicine, University of Washington, Seattle, WA USA; 18 Department of Global Health, University of Washington, Seattle, WA USA; 19 Present Address: Department of Microbiology, The Icahn School of Medicine at Mount Sinai, New York, NY USA

**Keywords:** Viral genetics, Sequencing, HIV infections

## Abstract

In the antibody mediated prevention (AMP) trials, the broadly neutralizing antibody (bNAb) VRC01 demonstrated protective efficacy against susceptible HIV strains. To understand how VRC01 shaped breakthrough infections, deep sequencing was performed on 172 participants (>100,000 *gag-Δpol* and *rev-env-Δnef* sequences), at diagnosis and over time, in the placebo and treatment arms of the African (HVTN703/HPTN081; NCT02568215) and Americas/Europe (HVTN704/HPTN085; NCT02716675) cohorts. A high frequency of multilineage infections was detected (38%), including co-infection with both VRC01 sensitive and resistant viruses. This high frequency is largely accounted for by low-abundance lineages. Although VRC01 does not significantly affect the genetic transmission bottleneck compared to placebo, higher VRC01 doses trend towards greater VRC01 neutralization differences among co-infecting lineages. Two-thirds of multilineage infections showed evidence of recombination at the diagnostic timepoint. In the treatment group there is evidence of recombinant viruses preferentially inheriting resistance-associated mutations. This study provides critical insights into viral genetic and antigenic diversity that needs to be targeted to achieve protection, and highlights the role of recombination in facilitating escape.

## Abstract (web summary)

Antibody mediated prevention (AMP) trials with the broadly neutralizing antibody VRC01 showed protection against VRC01-sensitive viruses. Here, by deep sequencing plasma samples from 172 participants of the AMP trials, the authors show a high frequency of multilineage HIV infections (38%), including coinfection with both sensitive and resistant viruses, and demonstrate that VRC01 doesn’t alter the transmission bottleneck.

## Introduction

HIV remains a significant global public health concern, and utilizing broadly neutralizing antibodies (bNAbs) for prevention is a crucial component of the strategy to combat its spread. The only efficacy studies assessing this concept, the antibody mediated prevention (AMP) trials, evaluated the effectiveness of the bNAb VRC01 (10 doses given every 8 weeks intravenously at either 10 mg/kg or 30 mg/kg) to prevent HIV acquisition. These trials were conducted in women in sub-Saharan Africa (HVTN 703/HPTN 081), and in cismen and transgender persons who have sex with men in the Americas and Europe (HVTN 704/HPTN 085)^1^. The AMP trials showed that VRC01 did not provide protection against overall HIV diagnosis, however it had 75% prevention efficacy against viruses sensitive to VRC01 (IC80 < 1 µg/ml)^1^. Viruses acquired in the VRC01 groups were more resistant to neutralization by the antibody than those in the placebo group (geometric mean IC80 of 8.4 µg/ml compared to 3.5 µg/ml, respectively) and exhibited greater divergence in the VRC01 epitope^1,2^. Together, these findings underscore the dynamic interplay between HIV and bNAbs and the need to overcome HIV resistance to achieve durable prevention and therapeutic/cure outcomes. To this end, combinations of bNAbs with enhanced breath and potency are being, or have been, evaluated in clinical trials^3–11^.

In people living with HIV, the virus persists as genetically related but evolving and diversifying genome populations, also known as “quasispecies”, although only one to a few virions typically establish clinical infection^12–16^. A meta-analysis of 70 studies estimated that 25% of individuals acquire multiple founder viruses, with higher rates in men who have sex with men (~30%) than in women (~21%)^17^. Following transmission, there is a selection bias for fitter viruses^18^ which preferentially use the CCR5 co-receptor and tend to be more interferon-alpha resistant^19–21^. In contrast, factors that disrupt the mucosal barrier, such as other sexually transmitted infections or genital inflammation, reduce this selection bias, resulting in a greater number of viruses establishing infection, including viral strains with lower infectivity^18,22–24^. Co-acquisition of phenotypically distinct variants has been reported and may occur either stochastically through the carryover of donor-adapted variants that are sometimes less fit in the new host, or due to transmission advantages^21,25–27^. Deep sequencing studies have also shown that low-abundance, drug-resistant variants can be transmitted to treatment-naïve individuals^28^.

In this study, we investigated whether the broadly neutralizing antibody VRC01, used in HIV prevention, influenced the transmission bottleneck and quasispecies composition following acquisition. To answer this question, we generated over 100,000 *gag-Δpol* (GP) and *rev-env-Δnef* (REN) sequences from participants in the AMP trials using a highly accurate PacBio SMRT-UMI deep sequencing protocol^29^. Because it is not possible to determine precisely which variants within a closely related population crossed the mucosal/blood barrier to establish infection, in this paper we refer to the viral populations that emerge after acquisition as transmitted founder lineages (TFL)^30^. By integrating sequence data with the estimated date of acquisition, predicted antibody concentrations and VRC01 neutralization sensitivities, this study defines virological outcomes of antibody-based pre-exposure prophylaxis (PrEP), and provides key insights into the challenges facing biomedical prevention and cure strategies.

## Results

### Description of participants and data generated

AMP trial participants were infused for 10 visits, every 8 weeks, with either 10 mg/kg VRC01, 30 mg/kg VRC01, or saline (placebo). Individuals were included in this study if they were diagnosed with HIV by the week 80 visit (primary endpoints) (Table 1). Those who acquired HIV after the week 80 visit were excluded due to declining VRC01 levels at the time of diagnosis. Individuals were tested for HIV acquisition every 4 weeks, and sequencing was performed on the first HIV RNA-positive plasma sample. In 159 individuals, at least one additional sample was sequenced - collected within a median of 13 days (IQR 6–21) after the first sequencing time point.

**Table 1:** Genotypic and neutralization characteristics of viruses in participants with HIV diagnosis by week 80 visit (primary endpoint)

	HVTN 703/HPTN 081 (African Trial)				HVTN 704/HPTN 085 (Americas Trial)			
	Placebo	VRC01 10 mg/kg	VRC01 30 mg/kg	Total	Placebo	VRC01 10 mg/kg	VRC01 30 mg/kg	Total
No. of primary endpoints^a^	29	28	17	74	38	32	28	98
No. of REN multilineage infections	11	8	9	28	13	13	10	36
No. of GP multilineage infections	11	7	8	26	14	12	6	32
No. of multilineage infections (GP and/or REN) (%)	11 (38%)	8 (29%)	9 (53%)	28 (38%)	15 (39%)	13 (41%)	10 (36%)	38 (39%)
No. of multilineage infections with IC80 data from >1 lineage	6	6	5	17	9	8	6	23
No. of VRC01 discordant multilineage infections^b^	1 (17%)	3 (50%)	2 (40%)	6 (35%)	3 (33%)	3 (38%)	4 (67%)	11 (48%)
No. of REN sequences first timepoint: mean (range)	134 (6–493)	115 (4–550)	143 (6–625)	129 (4–625)	175 (4–840)	148 (2–635)	117 (1–381)	150 (1–840)
No. of GP sequences first timepoint: mean (range)	117 (6–474)	101 (3–374)	141 (11–981)	116 (3–981)	159 (26–377)	180 (1–1055)	113 (6–235)	151 (1–1055)
No. of REN sequences second timepoint: mean (range)	123 (3–353)	168 (14–546)	223 (30–633)	163 (6–633)	149 (38–83)	224 (2–1128)	164 (31–421)	178 (2–1128)
No. of GP sequences second timepoint: mean (range)	167 (9–526)	211 (22–701)	227 (10–814)	199 (9–814)	145 (24–396)	150 (4–455)	107 (1–267)	136 (4–455)

^a^Primary endpoints include those participants with available first RNA+ sample sequencing data. The total endpoints in the Africa trial were 76, and two were excluded.

^b^Pseudoviruses where at least one pair were >3-fold difference in IC80 titers. REN: rev-env-∆nef. GP: gag-∆pol.

Long-read, deep sequencing data were generated from 172 individuals who acquired HIV in AMP: 74 from the HVTN 703/HPTN 081 Africa trial and 98 from the HVTN 704/HPTN 085 Americas trial (Table 1). Sequence accuracy was improved by labeling each cDNA with unique molecular identifiers (UMIs), and collapsing UMIs to generate consensus sequences. The input cDNA per reaction was limited to fewer than 25 amplifiable copies to minimize recombination, and the application of the PORPID pipeline further increased data quality by discarding sequences containing erroneous UMIs, recombination artifacts, heteroduplexes, or early-cycle PCR errors^31^. Two viral genomic regions were sequenced at each time point: *gag-Δpol* (GP) (2.5 kb) and *rev-env-Δnef* (REN) (3 kb), resulting in a total of 57,798 GP and 53,088 REN sequences, with a mean of 162 GP and 147 REN sequences generated per participant per timepoint. Neutralization data were generated using gp160 *env* plasmids representative of transmitted lineages from 162 individuals: 72 individuals from the Africa trial, and 90 individuals from the Americas trial^32^.

### Lineage classification

The lineage classification approach developed by Mullins et al.^30^ was applied to our study. That study used SMRT-UMI PacBio deep sequencing data from two cohorts (RV217 and FRESH) in which HIV screening occurred twice weekly. This high-frequency sampling enabled a precise estimation of infection timing and as a result, a clear distinction between sequence changes associated with separate TFL, and those attributed to post-acquisition evolution within a lineage. This temporal resolution provided a strong foundation for developing a more accurate and robust approach to lineage determination.

Using data from AMP from both time points and genomic regions, HIV sequence populations were classified into lineages, referred to as transmitted founder lineages (TFLs), with each lineage assumed to represent a distinct acquisition genotype. Comparing the lineage classification in the first timepoint, after removal of recombinant and hypermutated sequences, the median intra-lineage DNA distance at the first time point was very low: 0.05% for REN (IQR 0.04 and 0.08) and 0.04% for GP (IQR 0.03–0.05), with a median inter-lineage DNA distance of 1.68% for REN (IQR 0.94 − 2.66%), and 1.00% for GP (IQR 0.68–1.47) (Supplementary Fig. S1). This intra-lineage diversity is consistent with diversity previously observed in acute/early infection^15,16^.

The date of acquisition was  estimated by calculating the date of diagnosable infection by a Bayesian procedure that combined sequencing data with diagnostic data from the first RNA PCR positive time point, and  subtracting 7 days (to account for the eclipse period^33^), decribed in detail by Rosssenkhan et al. Multilineage infections were not associated with longer infection times: the median estimated time from acquisition for multilineage infections was 36 days (IQR 25–45) and for single lineage infections was 34 days (IQR 25–39 days). The difference of 2 days in median time from acquisition between multilineage and single acquisitions was not significant by 2-way Wilcoxon test (*p* = 0.15, 95%CI on a true difference of 0–9 days).

### Transmission bottleneck

**Fig. 1:**
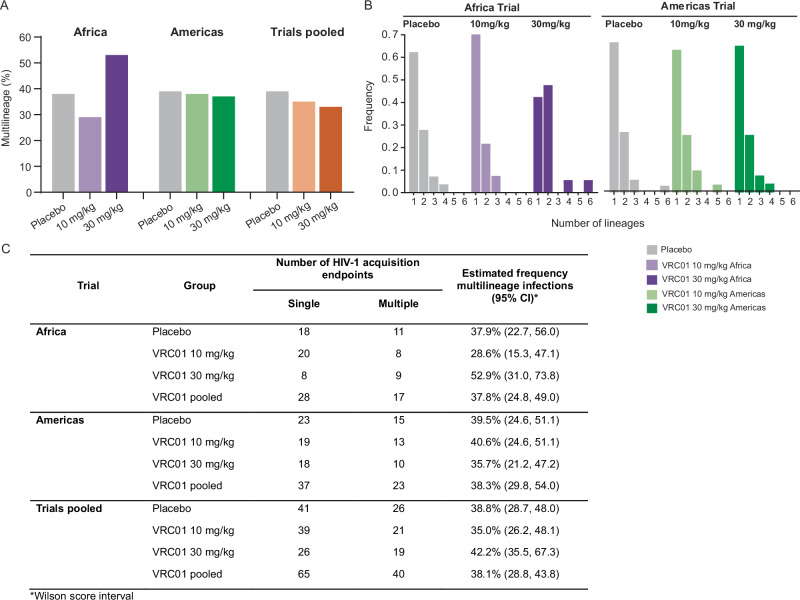
Frequency of multiple transmitted founder lineages (TFL) following HIV diagnosis across and within AMP trials. *Gag-Δpol* (GP) and *rev-env-Δnef* (REN) sequences were analyzed to identify individuals with multilineage infection, defined as the presence of more than one lineage in either gene region or at either sampling time point. **A** Bar graph comparing the percentage of multilineage infections across trial groups: Africa trial (purple); Americas trial (green); placebo gray; pooled trial analysis (orange). **B** Histogram illustrating the distribution of the number of REN lineages per individual, which ranged from 1 to 6. **C** Estimated frequencies of multilineage infections with Wilson score 95% confidence intervals. No statistical difference was found between groups within trials, or in pooled trial analyses.

We investigated if VRC01 treatment either restricted or relaxed the transmission bottleneck, and whether there was a difference between the trials. We compared the estimated frequencies of multilineage infections between trials, calculating Wilson score 95% confidence intervals (CI) for each estimate. (Fig. 1A, C). While differences in the VRC01 10 mg/kg (29%) and the 30 mg/kg (53%) groups were seen in the Africa trial, the differences were not statistically significant (Fig. 1C). No difference between the VRC01 groups combined and the placebo group (38%) was observed. In the Americas trial, the frequency of multilineage infection was very similar across all groups, with 41% and 36% of participants in the VRC01 10 mg/kg and 30 mg/kg groups, respectively, and 39% of participants in the placebo group (Fig. 1C). In addition, there was no difference in the frequency of multilineage infections between the placebo groups in the Africa and Americas trials (38% and 39%, respectively). Lastly, there was no difference between the placebo and VRC01 groups when dose groups were pooled within a study or when groups were pooled across studies.

Over 90% of individuals acquired three or fewer lineages. The number of TFLs was not different between treatment groups within each trial, nor between trials (Fig. 1B). The maximum number of lineages detected based on REN sequencing was six, and based on GP sequencing was nine (Supplementary Fig. S2).

### Factors contributing to higher-than-expected frequency of multilineage infections

**Fig. 2:**
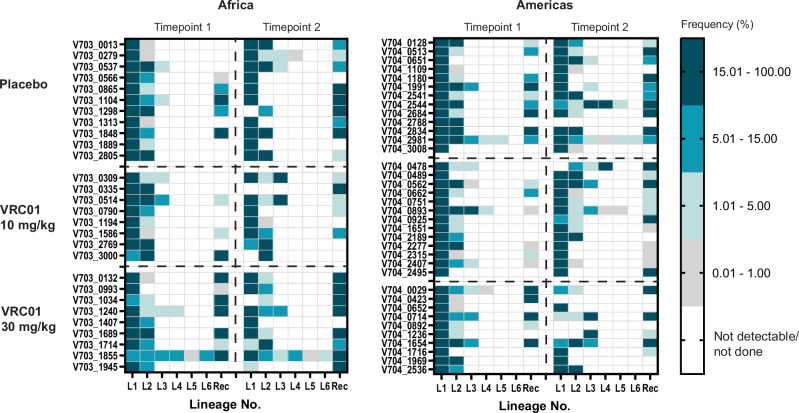
Heat plots illustrating the frequency (%) of *rev-env-Δnef* (REN) lineages (L1-6) and recombinant sequences (Rec), at two time points in the Africa and Americas trials. Time point 1 corresponds to the approximate time of HIV diagnosis, and time point 2 was sampled a median of 13 days (IQR 6–21 days) later. Each cell represents the percentage of sequences belonging to a specific lineage or recombinant form, colored from white (not detectable or not done) to dark teal (present in >15% of sequences).

The higher sequencing depth in the AMP trials was the major contributor to higher frequency of multilineage infections compared to previous studies^17^. With a sequencing depth of at least 100 copies, there is a 99% probability of detecting lineages present at a frequency of 5%, whereas a sequencing depth of 20 would miss these populations 36% of the time. Low-abundance viral populations, defined here as lineages comprising <5% of the total sequence count, were present in 20% of individuals (35/172 REN; 34/172 GP). Lineages present at a frequency of less than 1% were observed in 12 individuals, for both REN and GP (Fig. 2, Supplementary Fig. S2). AMP participants had an overall frequency of 38% multilineage infections. If minor lineages in REN were excluded, this number dropped to 22%, similar to the 25% estimated by Baxter et al.^17^. Similarly, following the removal of minor lineages in GP, only 23% of participants would have been determined to have multilineage infections (Supplementary Fig. S2).

Sequencing two timepoints also contributed to increased sensitivity, with multilineage infections detected in only one timepoint in 5.8% (10/172) of cases in REN and 3.5% (6/172) in GP. Sequencing two genomic regions made a minor contribution to higher frequency of multilineage infections: the overall frequency of multilineage infections in participants was 38%, with 37% of infections classified as multilineages by REN alone, and 34% by GP alone (Table 1). As noted by Mullins et al.^30^, discordant GP/REN multilineage classifications typically involved low abundance secondary lineages (median frequency <1%).

As VRC01 may suppress sensitive viral populations post-acquisition, we assessed whether there was an increased frequency of minor virus populations in the VRC01 arms (pooling across trials and treatment groups). There was no difference in the frequency of minor REN lineages among the individuals in the placebo group (24%; 16/67) compared to the VRC01 group (20%; 21/105) (Fisher’s exact test two sided: *p* = 0.57). The frequency of minor GP lineages in the placebo group was lower in the VRC01 group (14%; 15/105) compared to those in REN (25%; 17/67), with a trend toward significance (Fisher’s exact test two sided: *p* = 0.07)

Recombination was pervasive, as revealed in individuals with multilineage infections. At the first time point, recombinants were detected in REN in 62% of these individuals in the Africa trial, and 64% in the Americas trial, and in 69% and 66% of individuals, respectively, in GP. Pooling across trials and groups, the mean number of unique recombinants generated per day, among all participants for whom recombinants were detected was 0.65 (range 0.02–7.65) in REN and 0.48 (range 0.02–2.33) in GP.

### VRC01 discordant phenotypes in participants with HIV multilineage infections

VRC01 neutralization sensitivity was assessed using pseudoviruses generated from representative sequences of each TFL^1,32^. In single-lineage infections, viruses from the pooled VRC01 group (*n* = 65) were significantly more resistant to VRC01 than those from the placebo group (*n* = 41) (Mann-Whitney test: *p* < 0.001) (Supplementary Fig. S3).

**Fig. 3:**
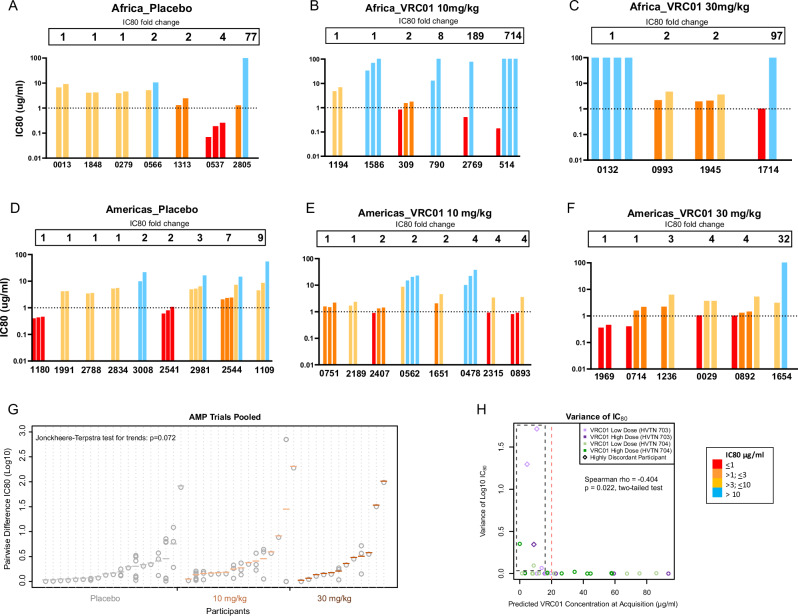
VRC01 discordant phenotypes in AMP participants with multilineage HIV infection. IC80 data shown for participants with neutralization data from multiple clones derived from the first time point. **A**–**C** Africa trial and **D**–**F** Americas trial: each bar corresponds to one virus from the participant identified by the 4 digit code below the graph. Fold change between the lowest and highest IC80 shown above the bars. **G** Log IC80 difference between placebo and VRC01 groups (trials pooled). Gray circles show individual pairwise IC80 differences, with colored horizontal lines showing the mean pairwise difference placebo (dark gray), VRC01 10 mg/kg (light brown), VRC01 30 mg/kg (burnt orange). Participants with two lineages have one measurement; those with ≥3 have more. Sorted by mean pairwise IC80 difference. Statistical significance determined with one-sided Jonckheere-Terpstra test for trends. **H** Variance in IC80 of all sequences (y-axis) vs. predicted VRC01 concentration at acquisition (estimated date of diagnosable infection minus 7 days). Black dashed box highlights 7 participants with high IC80 variance and low predicted concentration, three of which are slightly above baseline. Above 20 µg/ml predicted concentration (vertical red dashed line), log10 IC80 values had little variability. Statistical significance determined with two-sided Spearman’s rank-order correlation test.

Of the 66 individuals with multilineage infections, 41 had more than one clone representative of the different lineages with corresponding VRC01 neutralization data. One of these participants (V703_1240, VRC01 group) was infected with two HIV strains (dual infection), which may have occurred from two different infecting partners. Of the remaining 40 infections, 40% (*n* = 16) had pseudoviruses with greater than a 3-fold difference in their VRC01 IC80 titers (referred to as VRC01 discordant phenotype) (Table 2; Fig. 3A–F). VRC01 discordant phenotype infections at diagnosis were more frequent in the treatment group (11 of 24, 46%) compared to placebo (5 of 16, 31%), however, this difference was not statistically significant (Fisher’s exact two sided test: *p* = 0.51). Notably, three VRC01-treated individuals harbored both highly sensitive (IC80 < 1 µg/ml) and highly resistant (IC80 > 77 µg/ml) viruses at diagnosis, with differences in sensitivity ranging from 97- to 704-fold (Table 2). A trend of increasing intra-host neutralization differences was observed across placebo, low-dose, and high-dose VRC01 groups (Jonckheere-Terpstra test, *p* = 0.072; Fig. 3H). These findings suggest that an environment around the time of acquisition allowed both VRC01 sensitive and resistant viruses to co-exist.

**Table 2:** Maximum-likelihood pairwise DNA distances between and within a lineage of individuals who acquired viruses with VRC01 discordant phenotype (>3-fold difference in IC80) showing fold IC80 difference (*) and inter-lineage DNA distances (**) between the clones and their corresponding lineages with lowest and highest IC80 values

	PTID	No. lineages with IC80 data (total number lineages)	IC80 (mcg/mL)			DNA distance (mean)	
Treatment Group			Lowest	Highest	Fold difference*	Intra-lineage	Inter-lineage**
Placebo	V703_0537	3 (3)	0.07	0.26	4	0.03%	1.89%
	V703_2805	2 (2)	1.29	>100	78	0.03%	7.42%
	V704_1109	2 (2)	6.26	55.00	9	0.03%	1.84%
	V704_2544	3 (5)	2.06	14.77	7	0.02%	6.50%
	V704_2981	4 (6)	4.93	16.42	3	0.03%	2.42%
VRC01 10 mg/kg	V703_0790	2 (2)	13.02	>100	8	0.09%	0.28%
	V703_2769	2 (2)	0.41	77.56	189	0.05%	5.47%
	V703_0514	3 (3)	0.14	>100	714	0.07%	6.11%
	V704_0478	3 (3)	10.24	37.85	4	0.03%	1.30%
	V704_0893	3 (5)	0.81	3.58	4	0.07%	0.54%
	V704_2315	2 (2)	0.93	3.42	4	0.04%	1.43%
VRC01 30 mg/kg	V703_1714	2 (2)	1.03	>100	97	0.14%	1.25%
	V704_0029	2 (4)	1.06	3.72	4	0.06%	3.37%
	V704_0892	2 (2)	1.23	5.40	4	0.13%	1.02%
	V704_1236	2 (3)	2.25	6.38	3	0.03%	1.83%
	V704_1654	2 (3)	3.14	>100	32	0.07%	1.46%

We investigated the relationship between the lineages to determine if it was feasible that the VRC01 resistance phenotype in discordant virus infections could have arisen post-acquisition rather than being a property of the TFL (Table 2) (Supplementary Fig. S4). The median distance between lineages of 1.83% (IQR 1.25–2.70) exceeded the expected mutation rate for the duration of infection (median 25 days, IQR 18–41 days) (assuming an average mutation rate per nucleotide per generation cycle of 2.16 × 10^−5^)^34^. This suggests that the discordant phenotype is a property of the transmitted lineage rather than the result of post-acquisition evolution. There was one exception (participant V703_0790), who was infected for an estimated 45 days, and the proposed lineages differed by five distinguishing mutations. In this case, we could not exclude the possibility that resistance emerged after acquisition.

### Influence of VRC01 concentration on co-acquisition of sensitive and resistant phenotypes

We hypothesized that declining VRC01 antibody concentrations between infusions could allow a window of opportunity for co-acquisition of susceptible and resistant viral variants from an infecting partner. In this scenario, with relatively weak antibody pressure as antibody levels fell below the protective threshold, resistant viruses would still have a selective advantage while susceptible viruses would escape neutralization. To explore this possibility, the concentration of VRC01 at the estimated time of acquisition was predicted^35,36^ and compared to the variance of IC80s between lineages in the VRC01 groups (Fig. 3G). Overall, there was greater intra-participant variance in IC80s at lower predicted VRC01 concentrations (Spearman Rho = −0.404, *p* = 0.022). In contrast, when the predicted VRC01 concentration was >20 µg/ml, there was limited IC80 variability. The four individuals with the highest variance also had low predicted VRC01 concentrations at the estimated time of acquisition (<10.73 µg/ml) (V703_0514, V703_1714, V703_2769, V704_1654). Of these, three individuals in the VRC01 treatment groups acquired viruses that were highly sensitive (IC80 < 1 μg/ml) and highly resistant to VRC01 (IC80 > 70 μg/ml). The relationship between predicted VRC01 concentrations at estimated acquisition, and the proportion of resistant sequences was also analyzed (Supplementary Fig. S5A). Using IC80 > 3 μg/ml to define resistance, we observed a trend of increasing proportion of resistant sequences with increasing predicted concentrations of VRC01 (*p* = 0.054; Spearman Rho).

### Viral lineage growth kinetics in participants co-infected with VRC01 sensitivity discordant viruses

**Fig. 4:**
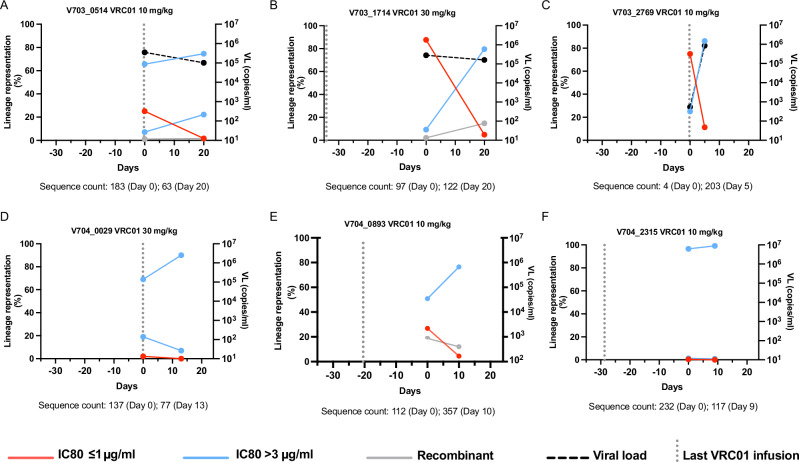
Lineage kinetics in participants who acquired both sensitive (red line) and resistant (blue line) viruses. **A**–**F** Lineages were assigned by the IC80 of their corresponding transmitted founder pseudovirus: IC80 < 1 red, IC80 > 3 blue, recombinant gray. Viral loads (VL) shown with segmented black lines, and the vertical gray dotted line indicates the timing of the last VRC01 infusion relative to day 0. Day 0 represents the first RNA positive timepoint, and the second timepoint is shown in days relative to day 0. Sequence count at each timepoint are indicated below each graph. The sampling interval ranged from 5-20 days Pseudovirus IC80 values: **A** V703_0514 IC80 > 100, >100, and 0.14 µg/ml; **B** V703_1714 IC80 > 100 and 1.03 µg/ml; **C** V703_2769 IC80 77.56 and 0.41 µg/ml; **D** V704_0029 IC80 3.72, 1.06 and 3.70 µg/ml (recombinant); **E**) V704_0893 IC80 3.58, 0.91 and 0.81 µg/ml; **F** V704_2315 IC80 3.42 and 0.93 µg/ml.

Six individuals that acquired virus isolates with an IC80 < 1 µg/ml (sensitive) and >3 µg/ml (resistant) had sequences derived from two timepoints (Fig. 4). Sequences within a lineage were designated as VRC01-sensitive or -resistant genotypes based on their genetic relatedness to the pseudovirus evaluated in the neutralization assay. We also screened sequences from these individuals for evidence of evolution in the VRC01 epitope^37–42^.

In four of the six participants (V703_0514, V703_1714, V703_2769 and V704_0893), the sensitive lineages were found at reduced frequencies at the later time point (5–20 days later) (Fig. 4) and were found only at very low abundance at both time points in the remaining two participants (V704_0029, V704_2315). In three participants (V703_0514, V703_2769 and V704_0029), the last VRC01 infusion (dotted vertical lines in Fig. 4) was given on the day of diagnosis and the first sequencing timepoint, and in the remaining three participants the last infusion was given 25–30 days prior to diagnosis and sequencing.

The relative frequency of each infecting lineage was plotted (Fig. 4) (sensitive red, resistant blue, recombinant gray). In one participant (V704_0029) the lineages were at very low abundance with recombinants accounting for 69% and 90% of the sequences sampled at the first and second time points, respectively. Mutations at the 279 site, located in the Loop D VRC01 contact site^4,37,38,41,43^, were observed in 9 sequences, one sampled at the first time point, and the rest at the second time point (3% of the total 190 sequences). All sequences were recombinants (Wald–Wolfowitz runs test *p* values ranging between 0.01 and 10^−5^). Five of the 9 sequences were direct recombinants of the TFLs, and since both parental lineages contained the wildtype D279 variant, it appears the A/E/H/N mutations at position 279 arose after viral acquisition in a recombinant backbone. However, once a resistance-associated mutation appeared, it was propagated through recombination, as all of the remaining 4 recombinant sequences carrying any of the mutations away from D279 had parental strains discordant at site 279 (one parent carried D279 and the other carried the mutated variant), and yet all recombinant daughters inherited the resistance mutations (binomial *p* = 0.1; Supplementary Fig. S6).

**Fig. 5:**
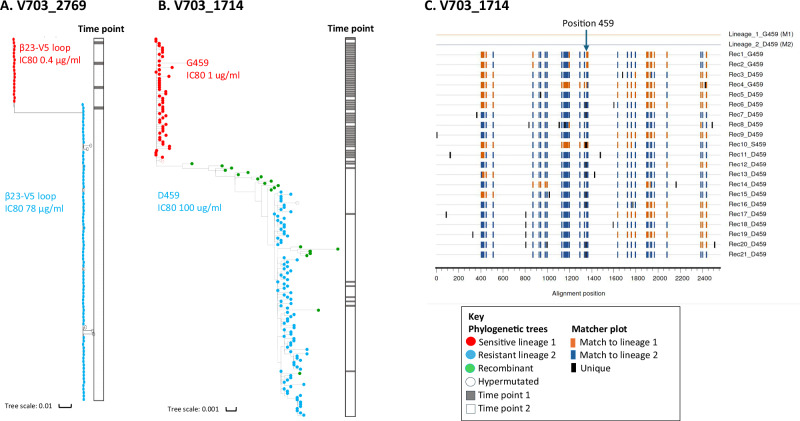
Sequence evolution in two individuals co-infected with VRC01 highly sensitive (IC80 < 1 μg/ml) and resistant (IC80 > 70 μg/ml) lineages. In both individuals, amino acids conferring VRC01 resistance were located in the β23-V5 loop^44^. Phylogenetic trees of env sequences from V703_2769 (**A**) and V703_1714 (**B**) show sequences with amino acids conferring VRC01 sensitive mutations (red dots), resistant mutations (blue dots), and recombinants (green dots). Sequences were obtained from 2 sample time points: 5 days apart for V703_2769, and 20 days apart for V703_1714. The ribbon on the right of each tree illustrates sampling times: gray for time points 1 and white for time point 2. **C** Matches Highlighter plot (https://www.hiv.lanl.gov/content/sequence/HIGHLIGHT/highlighter_top.html) for V703_1714 showing all recombinant sequences in comparison to the two TFL parental sequences at the top. Each row represents a single env sequence, annotated with either G459 (sensitive) or D459 (resistance) mutations. Vertical tick marks indicate site associated with the 2 lineages: orange for lineage 1, dark blue for lineage 2, and black for unique changes. The arrow marks the HXB2 position 459 with partial resistance mapped to the G459D mutation. All recombinant sequences were screened using the LANL tool RAPR and yielded recombination *p* values between 10x^−9^ and 0.007 by Wald–Wolfowitz runs test. While the parental lineages had discordant genotypes at position 459 (one sensitive, one resistant to VRC01), all recombinant daughters carried the resistance-inferring mutation (binomial *p* = 3 × 10^−5^).

Resistance was experimentally defined in two of the six individuals with multilineage infections (V703_2769 and V703_1714), allowing for detailed analysis of lineage dynamics^44^. Participant V703_2769 was infected with viruses with IC80s of 0.4 µg/ml and 78 µg/ml, and resistance was mapped to changes in the β23-V5 loop (Fig. 5A). Participant V703_1714 was infected with viruses with an IC80 of 1.0 µg/ml and >100 µg/ml with partial resistance mapped to the G459D mutation (Fig. 5B). The abundance of sequences with sensitive mutations declined over the 5-day sampling interval for V703_2769, and over the 20-day interval for V703_1714, along with a concomitant increase in abundance of sequences with resistance mutations (Fig. 5A, B). Recombination was observed in V703_1714 (Fig. 5B, C) with two inter-lineage recombinants (2% of sequences) at the first time point and 18 (15% of sequences) at the second time point (Wald–Wolfowitz runs test recombination *p* values between 10^−9^ and 0.007). Of these, two early and 14 later recombinants were direct descendants of the two primary lineages, and all inherited the resistance-conferring amino acid D459 - indicating that recombination favored the resistant genotype (binomial *p* = 3 × 10^−5^).

## Discussion

Long-read deep sequencing made it possible to characterize the impact of VRC01 on breakthrough infections at a high-resolution. This level of sensitivity, with over 100,000 sequences generated from two genomic regions, was crucial for detecting subtle acquisition and post-acquisition effects that were not identified in previous sieve analysis based on the evaluation of a single sequence per lineage^2^. In the Africa trial, nearly all infections were subtype C and occurred among women with a predominantly heterosexual exposure risk. In contrast, in the Americas trial, infections were predominantly with subtypes B and F and BF recombinants, and occurred among men who have sex with men and transgender participants with a predominantly homosexual exposure risk^1,4^. We observed no differences in the frequency of multilineage infection comparing placebo and treatment groups within each trial, and also no difference when comparing placebo and treatment groups between trials. The overall frequency of 38% across all 172 acquisitions was 1.3–1.9 fold higher than previously reported, which used approaches with limited sampling depth^17^. Despite recognition of this ‘looser’ bottleneck, acquisition of viral populations remained highly constrained, with three or fewer lineages detected in 90% of participants.

Defining TFL using deep sequencing permits sensitive detection of founder viral quasispecies, yet distinguishing amino acid changes attributed to founder lineages versus those attributed to evolution post-acquisition remains challenging. Leveraging the unique designs of the RV217 and FRESH cohorts, where a precise time of HIV acquisition was defined by twice-weekly HIV screening, Mullins et al.^30^, developed methodologies, applied in our study, to classify single versus multi lineage infections and provided the foundation for our analysis investigating the effect of VRC01 on breakthrough infections.

Deep sequencing revealed that low-frequency viral lineages (<5% by abundance) were relatively common, detected in ~20% of individuals in both the placebo and VRC01 groups, and were the major contributor to the higher frequency of multilineage acquisitions identified, as noted by Mullins et al.^30^. Multilineage infection allows more rapid immune evasion post-acquisition through recombination, which is a more frequent event per viral replication cycle compared to point mutations^45^. Indeed, in this study recombination was observed in early infection in over 60% of individuals with multilineage infections. From a pathogenesis perspective, high viral diversity including multiple founder infections, has been associated with faster disease progression^46–48^. This would need to be re-evaluated in light of the prevalence of minor lineages not detectable or considered in previous studies. The reasons for the low abundance of some lineages are unclear, with possible influences including viral fitness, host immunity (innate or possibly adaptive), availability of target cells or stochastic effects during early seeding and viral population growth.

The AMP trial showed that VRC01 selected for resistant viruses^1^. Consistent with this, single-lineage infections in the VRC01 treatment group were more resistant compared to those in the placebo group. However, there was evidence of a differential impact of VRC01 on multilineage acquisitions, where 40% were infected with viruses that differed in VRC01 sensitivity (IC80 > 3-fold difference). Higher predicted VRC01 concentrations at acquisition trended to select resistant viruses (IC80 > 3 µg/ml), suggesting it is possible that waning levels of VRC01 between infusions enabled acquisition and outgrowth of highly sensitive viruses alongside resistant viruses. We hypothesized that there may be a threshold at which VRC01 levels are sufficient to select for resistant viruses, but were too low to fully prevent acquisition of sensitive ones. Indeed, greater variance in IC80 was observed at predicted VRC01 concentrations that were less than 20 µg/ml at acquisition. While statistically significant, this relationship may be stronger than indicated by the Spearman correlations and *p* values because the uncertainty in estimating VRC01 levels at acquisition reduces statistical power. The AMP trial used the TZM-bl assay, which is the standard method for measuring neutralization in clinical trials and has been shown to be a reliable biomarker of HIV prevention^35,49,50^. However, studies using replication-competent viruses have shown that higher amounts of CD4bs bNAb may be necessary for neutralization than what is measured by the TZM-bl assay^51^. Using different assays to study neutralization, including those that assess cell-to-cell transmission, could provide further insight into breakthrough infections with viruses that are classified as either sensitive or resistant in the TZM-bl assay.

Six individuals, all in the VRC01 treatment groups, acquired both highly sensitive (IC80 < 1 µg/ml) and resistant (IC80 > 3 µg/ml) viruses. This allowed the study of the effect of VRC01 in an environment where viruses with different VRC01 sensitivities replicated under similar conditions. We found a decrease in proportion of sensitive viruses over time with a concomitant increase in resistant ones, suggesting that VRC01 levels post-acquisition differentially suppressed viral populations post-acquisition, and providing an environment where resistant viruses had a selective advantage. Recombination was pervasive and in two examples, one where resistance mutation was mapped^44^, emerging recombinant viruses were more likely to inherit the VRC01-resistant mutation.

The high frequency of multilineage infections has importance for HIV pathogenesis, as well as HIV reservoir/cure and prevention strategies. Deep sequencing revealed that a high proportion of breakthrough infections are founded by multiple genetically distinct lineages in both the placebo and antibody-treated recipients, suggesting that this variation is an inherent feature of HIV transmission. These viral features expand the spectrum of viral targets that need to be blocked to achieve protection, likely increasing both potency and breadth required for immunological approaches to cure and prevention to be maximally effective. Current assessments of antibody breadth typically rely on panels representing dominant transmitted lineages and, as such, do not reflect the extent of phenotypic and genotypic diversity identified in this study. Our findings underscore the importance of considering the full viral population, rather than single representative strains from participants/donors, when assessing bNAb or vaccine targets. The AMP viruses represent a valuable contemporary resource for improving these panels^4^ and our data suggest that the methods utilized by us should be employed more frequently in collecting and assessing newly diagnosed HIV infection in population based studies of HIV diversity and evolution.

Our study supports that there is not a sharp cutoff in potency of a single bNAb to HIV and that VRC01 was able to reduce acquisition of very sensitive viruses. But in addition, some resistant variants may emerge under selective antibody pressure—as evidenced in follow-up sequencing, usually obtained within 2 weeks of the initial sample and show alterations in their genetic profile and in vitro testing, suggesting escape to VRC01. This was perhaps, in retrospect, inevitable in that the tail end of the PK profile of 30 mg/kg of VRC01 delivered a median predicted neutralization titer (PT80 or predictive titer corresponding to 80% neutralization of a given viral strain) against exposing viruses of 5.4 over the last 4 weeks post-infusion, well below the PT80 of 194 threshold required for high grade protection.

This emphasizes the importance of using more than one bNAb, especially combinations with complementary sites of activity to reduce the likelihood of emergence of clinically relevant antibody escape. Although preventing acquisition differs from suppressing resurgent viral replication, important shared lessons emerge. A key concern in PrEP interventions is the emergence of resistance during the pharmacokinetic “tail”, when product concentrations wane to below protective levels but are at sufficient concentrations to exert selective pressure on the virus^52^. As seen with Cabotegravir^53^. It remains unclear whether other long-acting agents, such as lenacapavir, which has shown near-complete protection against HIV acquisition, will be vulnerable to developing post-acquisition resistance. Although no resistance to lenacapavir has been reported in prevention trials^54,55^, it has been identified in treatment settings^56,57^.

Similarly, in analytical treatment interruption (ATIs) trials, resistance has emerged in combination bNAb treatment when levels of one bNAb decline below therapeutic thresholds, effectively resulting in montherapy^3,58^. In contrast, in a setting more analogous to prevention, where treatment was initiated in hyper-acute infection (RNA-positive, antibody-negative) and viral diversity was constrained by the transmission bottleneck, VRC01 administration followed by ATI was not associated with VRC01-driven *env* evolution or changes in VRC01 sensitivity^59^. Although low viral diversity in this setting may have constrained escape, PrEP studies demonstrate that resistance can still emerge when selective pressure is applied after acquisition, seen here with a single bNAb and previously with antiretroviral agents^53^. Moreover, multilineage infections can accelerate viral evolution through recombination, facilitating the emergence of escape variants, as was observed in this study. Quantitating the ability of new combination bNAbs to reduce transmission or reservoir reactivation needs to be further evaluated.

Our findings provide important insights into the challenges and future optimization of antibody-based PrEP. The success of next-generation bNAb-based PrEP approaches will depend on the breadth, potency, serum half-life, and biodistribution of the antibodies used^3,6,49,60–64^. The genomic and analytical tools used in this study provide a powerful framework to investigate whether combination prevention approaches can limit post-acquisition selection and improve durability of protection against HIV acquisition.

## Methods

### Ethical statement

All work described here complied with all relevant ethical regulations. The Institutional Review Boards/Ethic Committees of participating clinical research sites approved the studies, which were conducted under the oversight of the NIAID Data Safety Monitoring Board^1^. All participants gave written informed consent. The antibody neutralization assays were approved by the Duke University Health System Institutional Review Board (Duke University) through protocol no. Pro00093087 and the University of the Witwatersrand Human Research Ethics Committee (protocol no. M201105). Viral genome sequencing at the University of Cape Town was approved by the UCT Human Research Ethics Committee (HREC reference no. 176/2017) and was considered exempt at the University of Washington.

### Study populations

HVTN 703/HPTN 081, referred to as the Africa trial, enrolled 1,924 women with a high likelihood of HIV acquisition in Botswana, Kenya, Malawi, Mozambique, South Africa, Tanzania, and Zimbabwe. HVTN 704/HPTN 085, referred to as the Americas trial, enrolled 2,704 men who have sex with men with a high likelihood of HIV acquisition from Brazil, Peru, Switzerland, and the United States. Participants were randomized and given infusions of either 10 mg/kg VRC01, 30 mg/kg VRC01, or saline (placebo) (1:1:1 ratio) every 8 weeks, with HIV testing scheduled at 4 week intervals^1^. Positive HIV RNA diagnosis was confirmed with a second independent sample. The last infusion was scheduled at the week 72 visit, and the primary study endpoints included participants who were diagnosed by the week 80 visit. This report only includes individuals who met the primary endpoints.

### Viral genome sequencing

Sequencing was performed using the Pacific Biosciences single molecule real-time approach with unique molecular identifiers (SMRT-UMI) as described^29^. In brief, during HIV cDNA synthesis, each viral genome was tagged with a UMI, followed by amplification of an estimated 100 or more templates corresponding to the *gag*-Δ*pol* (GP) genomic region (2.5 kb; HXB2 positions 790–2292) and the *rev-env-*Δ*nef* (REN) region (3 kb; HXB2 positions 5970 – 9012). Distinct primer sets were used for the HVTN 703 and 704 trials (Supplementary Table 1). The number of amplifiable copies was estimated by end-point dilution using the QUALITY program to estimate the amplifiable templates (https://indra.mullins.microbiol.washington.edu/quality/). We targeted at least 100 sequences per sample, and due to high quantitation error, between 200 and 250 templates were amplified. Low viral load samples (less than 5000 copies/mL) were not quantified. Instead, cDNA from up to 1 mL of plasma was split into 20 nested PCRs. Only samples where the amplicon was visualized on the gel were processed by SMRT-UMI. Amplicons were sequenced on the PacBio platform using Sequel and Sequel II instruments. Circular consensus sequences were generated, and sequence data processed using a custom bioinformatics pipeline, PORPID pipeline (http://github.com/MurrellGroup/PORPIDpipeline)^29^. Sequence accuracy was enhanced by collapsing UMIs to generate a consensus sequence and limiting input cDNA molecules to minimize recombination (<25 amplifiable copies of cDNA). The PORPID pipeline discards sequences with erroneous UMIs, recombinants, heteroduplexes, or sequences with early-cycle PCR errors. Using this protocol, Westfall et al.^29^ estimated PCR and sequencing error rates to be less than 8.63 × 10^−8^ per base, or less than one error in every ~3700 REN sequences.

Nucleotide and amino acid alignments were generated using Muscle5^65^ and refined using Geneious (https://www.geneious.com) as described^30^ Participants in the Africa trial predominantly acquired subtype C (98%), with one G and one A1C recombinant identified^4,32^. Participants in the Americas trial predominantly acquired subtype B (73%), subtype F (11%), and recombinant B/F (11%), with subtype C and D, and recombinant forms A1/B, B/D and CRF47_BF also detected^4,32^.

### Lineage determination

Lineage assignment is described in detail by Mullins et al.^30^. Briefly, two major approaches were taken: the poisson fitter method^66^ applied to the first sequencing time point only, and a phylogenetic/distance method which analyzed data from all timepoints. The distance/phylogenetic approach generated maximum-likelihood phylogenetic trees from all timepoints pooled. Nucleotide changes from the most common sequence at the first time point were visualized using highlighter and matcher plots in Phylobook (https://github.com/MullinsLab/phylobook)^67^. Trees with single clades with low pairwise distances were classified as single lineage infections. Seqeunces were classified as  multiple lineage infections if there was more than one clade on the phylogenetic trees with inter-clade distances, after exclusion of recombinant and hypermutated sequences, greater than the intra-clade distances. Lineage assignment was supported by k-medoid clustering, the, and gap-methods^68^. Hypermutated sequences were identified using the LANL Hypermut tool (https://www.hiv.lanl.gov/HYPERMUT). Visual inspection using Phylobook^67^ and the Recombination Analysis Program (RAPR, https://www.hiv.lanl.gov/RAP2017) were used to screen for recombinants (the latter using a significance threshold of FDR *q* = 0.2)^69^. Scattered mutations were ignored and the minimum number of shared nucleotide changes to define a lineage for both GP and REN was four, with a minimum of two shared changes across a lineage used to define a recombinant. These cut-offs were defined using sequences generated using the same protocol, from participants from the FRESH^70^ and RV217^71^ cohorts with short diagnostic intervals (median 4 days between the last negative and first positive RNA tests)^30^. Lineage classification was reviewed by at least four investigators from different institution. Infections were classified as multilineage if the additional lineage was supported by more than one sequence, or if there was evidence of persistence in recombinant forms. Maximum likelihood pairwise distance distributions were generated using PhyML v3.3^72^ within DIVEIN^73^.

To calculate recombination rates, statistical support for each unique recombination event was obtained using the Wald–Wolfowitz Runs Test^69^ implemented in the LANL tool RAPR (https://www.hiv.lanl.gov/content/sequence/RAP2017/rap.html), with the most common sequence in each distinct lineage as a potential parental strain. In participants with VRC01 discordant phenotypes, RAPR was used to estimate how recombination affected the prevalence of the escape/resistance mutations. This included sequences from all available time points and a multiple testing FDR threshold of *q* < 0.2. The binomial test was used to determine whether resistance mutations were favored by recombination across recombination events where the parental strains carried discordant genotypes. For this test, the null hypothesis is that by chance alone, we expected each discordant genotype to appear in the recombinant daughter 50% of the times.

### Pseudovirus production and neutralization assays

For identification of sequences for synthesis and cloning, all samples were initially subjected to Sanger sequencing and if homogeneous, the *rev-env* sequence from that variant was synthesized for neutralization testing. Alternatively, cDNA was diluted to end-point to generate sequences from single viral templates to identify the major variants present^1^. All samples were subsequently also sequenced using the (SMRT-UMI) PacBio platform, and representatives of additional lower frequency lineages were synthesized and cloned. The *rev-env* portion of sequences representative of the different viral lineages were synthesized and cloned and the sequence reverified. 293 T/17 cells were co-transfected with *env* plasmids and an *env*-deleted backbone vector (pSG3delEnv). Pseudoviruses were then titrated to determine the optimal dilutions to use in the TZM-bl neutralization assay^74^. Neutralization was measured as a function of reduction in luciferase reporter gene expression (due to the presence of bNAbs) after a single round of HIV Env-pseudotyped virus infection of TZM-bl target cells. Neutralization titerss are expressed as the concentration of VRC01 at which relative luminescence units (RLU) were reduced by 80% (IC80) relative to virus control wells after subtraction of background RLU in cell control wells.

### VRC01 discordant phenotypes

Intra-participant differences in IC80 were calculated as the fold change between the most sensitive and most resistant clones. Discordant phenotypes were defined when two clones were greater than 3-fold different in their IC80 values, a threshold which considers assay variability^50^. Clones classified as recombinant were excluded from this analysis. The calculation of estimated dates of diagnosable acquisition (EDDA) is described in detail by refs. ^35,36^. In summary, the EDDA was estimated using a Bayesian posterior distribution, which combined independent inputs from diagnostic timing estimators with combined gag-Δpol and *rev-env-Δnef* based estimators utilizing Poisson Fitter 2.0^66^. When two sequenced regions yielded distinct timing estimates (that is, the 95% posterior credible intervals did not overlap), the *gag-Δpol* region was chosen as the final time estimate as this region does not encode a protein targeted by VRC01. In this study, the date of acquisition was assumed to be 7 days prior to the EDDA to account for the infection eclipse phase. VRC01 serum concentrations at acquisition were predicted by a 2-compartment population pharmacokinetics (popPK) model^75,76^. To examine the relationship between VRC01 levels at acquisition and the probability of multiple TFLs, we correlated available IC80 values at the first sequencing timepoint with the participant’s predicted VRC01 concentration at the estimated time of acquisition.

### Statistical analysis

The probability of detecting low frequency lineages was modeled assuming random sampling of independent viral templates^16^. The probability of detecting a lineage present at frequency *p* after sequencing *n* templates is $P=1-{(1-p)}^{n}$. Two-tailed 95% CI for the frequency of multilineage compared to single lineage acquisitions were estimated by the Wilson score interval for binomial proportions. Distributions of IC80 values were evaluated for each primary endpoint case (placebo vs VRC01 dose groups and trials pooled) using the Mann-Whitney *U* test (two-tailed). Intra-participant differences in VRC01 sensitivity were summarized by the mean of their pairwise-clone log10-transformed IC80 values. The individual mean values were then compared across groups and the three ordered treatment groups placebo, VRC01 10 mg/kg, VRC01 30 mg/kg using the Jonckheere-Terpstra test for trend (one-sided)^77^. All statistical tests examining the relationship between VRC01 levels at estimated acquisition and the probability of multiple TFL, including the analyses of intra-participant IC80 variance, and the recombination statistical tests (Runs Test and Binomial Tests) were done with correlation tests using the Spearman Rho coefficient (two-tailed *p* values). Fischer’s exact test (two-tailed) was used to determine if there was a significant association between frequencies of minor lineages or VRC01 discordant phenotype between treatment groups. Analyses were conducted in R version 4.3 (https://www.r-project.org/).

### Reporting summary

Further information on research design is available in the Nature Portfolio Reporting Summary linked to this article.

## Supplementary information

Supplementary Information

Peer Review file

Reporting Summary

## Data Availability

The neutralization and other data underlying the findings of this manuscript are publicly available at the public-facing HVTN data repository at Harvard Dataverse (10.7910/DVN/3VG3JO), in the files “v703_survival_wk80_tau_sieve.tab” and “v704_survival_wk80_tau_sieve.tab”. The data dictionary for these files is “README_survival.txt”. All individual participant data have been deidentified. All final GP and REN sequences were deposited in GenBank with Accession numbers: PX890939-PX026359; ON890939-ON891092; PX184492-PX150096 ON980967- ON980814; OR508960- OR508936; OQ912897-OQ912888.1. The GenBank accession numbers for the HIV Env clones used in the TZM-bl target cell neutralization assay are: HVTN 703/HPTN 081 sequences, ON890939–ON891092; HVTN 704/HPTN 085 sequences, ON980814–ON980967.

## Code availability

Some analyses required custom code written in R. This code is archived for public access at https://github.com/cmagaret/williamson_breakthrough_paper.
