# Supplementary material for: Influence of the broadly neutralizing antibody VRC01 on HIV breakthrough virus populations in antibody-mediated prevention trials
Source: Nat Commun. 2026 Apr 3;17:4780. doi: 10.1038/s41467-026-70888-0 (PMC13219488; doi:10.1038/s41467-026-70888-0)
Supplement: Supplementary file 1 — Supplementary Information [file 41467_2026_70888_MOESM1_ESM.pdf]

Supplementary Table 1: Primers used for cDNA synthesis and amplification of rev-env-nef (REN) and gag-pol (GP)

HVTN 704 primers

**Name**	**Usage**	**Sequence**
PB_AX_R9013	REN cDNA synthesis	**CCCGCGTGGCCTCCTGAATTAT***CCGCTCCGTCCGACGACTCACTATA* XXXXXX NNNNNNNNGTCATTGGTCTTAAAGGTACCTG
PB-R1-alt1	1st round PCR reverse primer	**CCCGCGTGGCCTCCTGAATTAT**
PB-R2-alt1	2nd round PCR reverse primer	*CCGCTCCGTCCGACGACTCACTATA*
F5876	REN 1st round PCR forward primer	TAGAGCCCTGGAAGCATCCAGGAAG
F5982	REN 2nd round PCR forward prime	GATCAAGCTTTAGGCATCTCCTATGGCAGGAAGAAG
R9013	REN 1st rd reverse primer for positive control	GTCATTGGTCTTAAAGGTACCTG
R8881	REN 2nd rd reverse primer for positive control	AGCTGGATCCGTCTCGAGATACTGCTCCCACCC
PB_AX_RT2	GP cDNA synthesis	**CCCGCGTGGCCTCCTGAATTAT***CCGCTCCGTCCGACGACTCACTATA* XXXXXX NNNNNNNNGTATGTCATTGACAGTCCAGC
F683	GP 1st round PCR forward primer	CTCTCGACGCAGGACTCGGCTTG
F762	GP 2nd round PCR forward prime	TTGACTAGCGGAGGCTAGAAGGAGA
RT2	GP 1st rd reverse primer for positive control	GTATGTCATTGACAGTCCAGC
RSP15R	GP 2nd rd reverse primer for positive control	CAATTCCCCCTATCATTTTTGGTTTCC

HVTN 703 primers

**Name**	**Usage**	**Sequence**
PB_A1_nef67_degen_PID_cDNA	REN cDNA synthesis	**CCCGCGTGGCCTCCTGAATTAT***CCGCTCCGTCCGACGACTCACTATA* XXXXXX NNNNNNNNGGTCTTAAAGGYACCTGAGGTCTGACTGGAAAGCC
F5876_SubC	REN 1st round PCR forward primer	TAGAGCCCTGGAACCATCCAGGAAG
pb_envArx_F	REN 2nd round PCR forward primer	GGCTTAGGCATCTCCTATAGCAGGAAGAA
EnvN	REN 1st rd reverse primer for positive control	TTGCCAATCAAGGAAGTAGCCTTGTGT
E01	REN 2nd rd reverse primer for positive control	TCCAGTCCCCCCTTTTCTTTTAAAAA
PB_AX_C6dn_PID_NIHCure	GP cDNA synthesis	**CCCGCGTGGCCTCCTGAATTAT***CCGCTCCGTCCGACGACTCACTATA* XXXXXX NNNNNNNNTGATGGGTCATAATATACTCCATG
Gag_D_F	GP 1st round PCR forward primer	TCTCTAGCAGTGGCGCCCG
Gag_A_F	GP 2nd round PCR forward prime	CTCTCGACGCAGGACTCGGCTT
07Rev6	GP 1st rd reverse primer for positive control	CTRTTAGCTGCCCCATCTACATAG
07Rev5	GP 2nd rd reverse primer for positive control	GTCCATTTRTCAGGATGGAGYTCAT
PB-R1-alt1	1st round PCR reverse primer	**CCCGCGTGGCCTCCTGAATTAT**
PB-R2-alt1	2nd round PCR reverse primer	*CCGCTCCGTCCGACGACTCACTATA*

Supplementary Figures

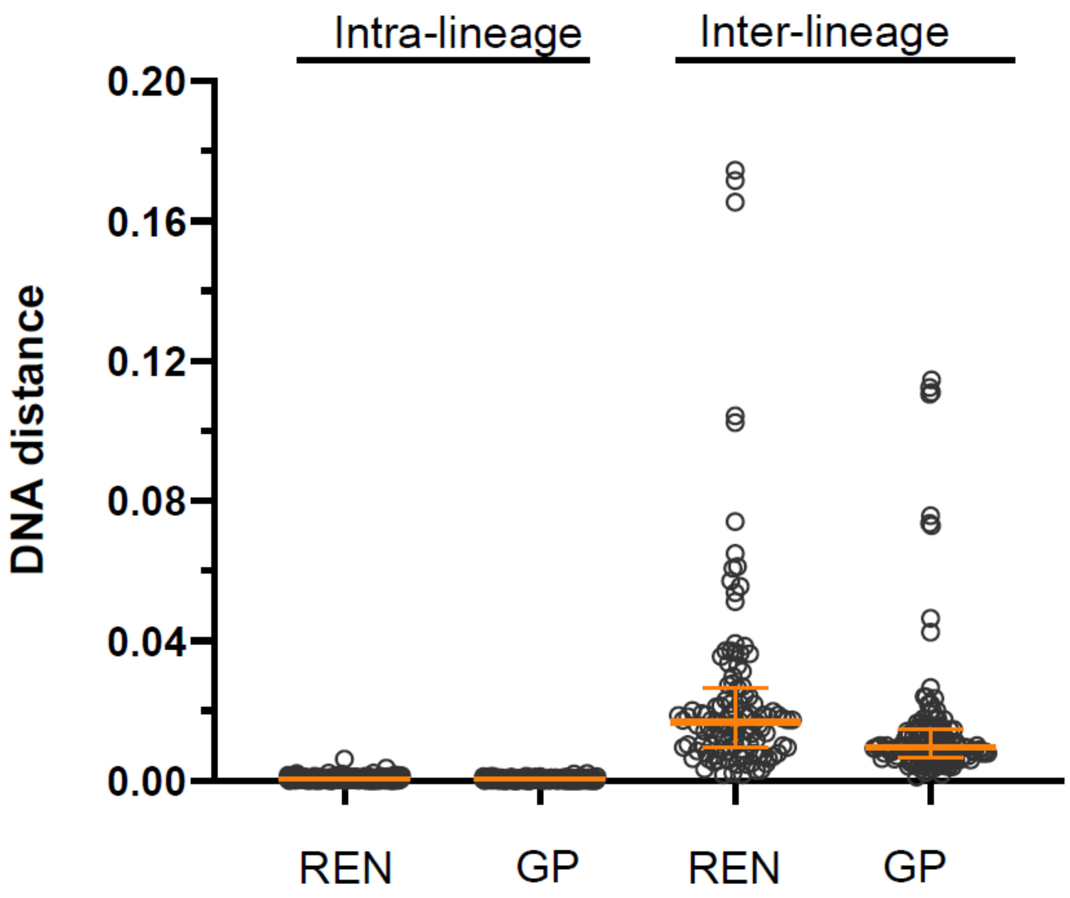

Figure S1. **REN and GP DNA distances.** Intra-lineage and inter-lineage DNA distances at the first sequencing time point only for all participants who met study endpoint. Participants included: V703_REN (n=74), V703_GP (n=63), V704_REN (n=97) and V704_GP (n=96). The upper and lower horizontal lines indicate the inter-quartile range and the middle horizontal line indicates the median.

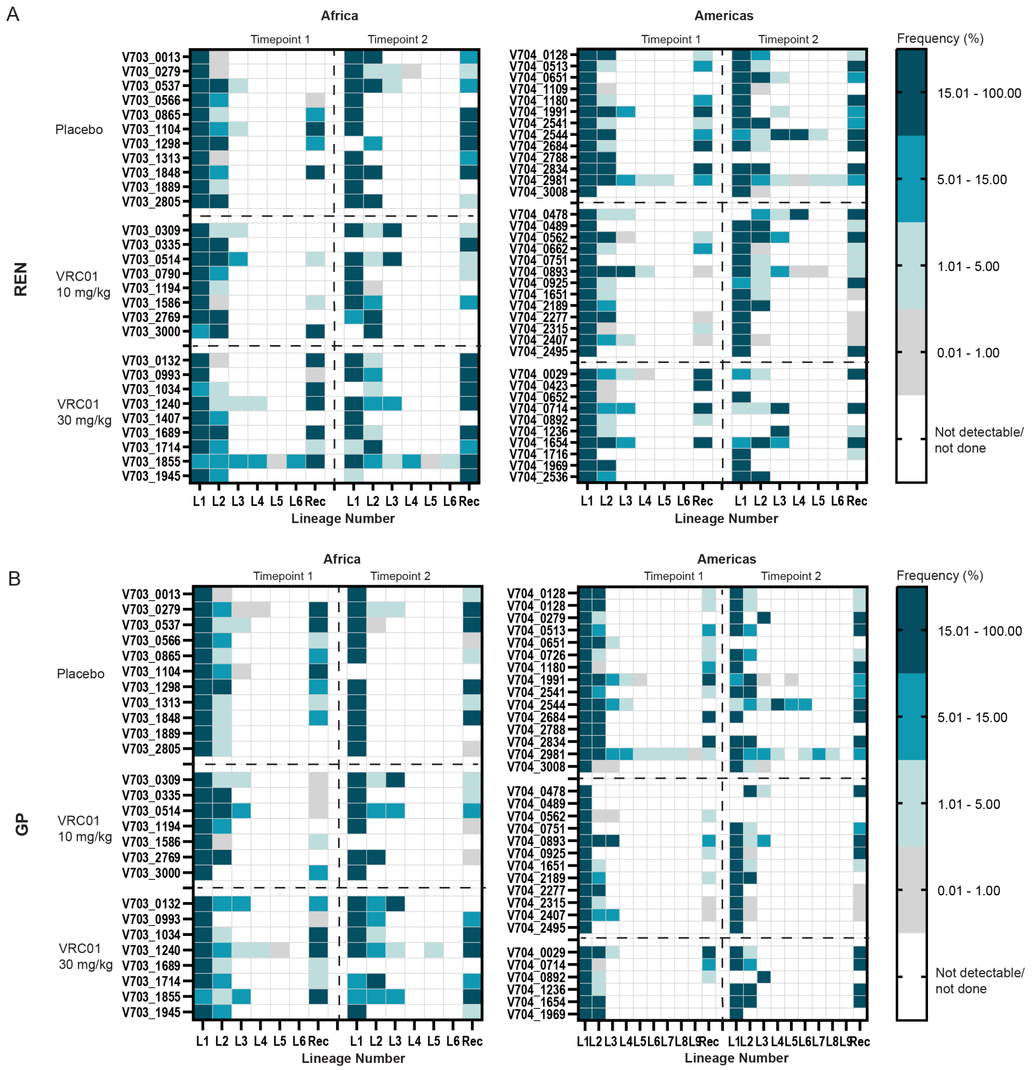

Figure S2. Heat plots illustrating the lineage frequency (%). A) rev-env-Δnef (REN) lineages (L1-6) and recombinant sequences (Rec), and **B)** Gag-Δpol (GP) (L1-9) at two time points in the Africa and Americas trials. Timepoint 1 corresponds to the approximate time of HIV diagnosis, and timepoint 2 was sampled on average of 17 days later (IQR 8-20 days). Each cell represents the percentage of sequences belonging to a specific lineage or recombinant form, colored from white (not detectable or not done) to dark teal (present in >15% of sequences).

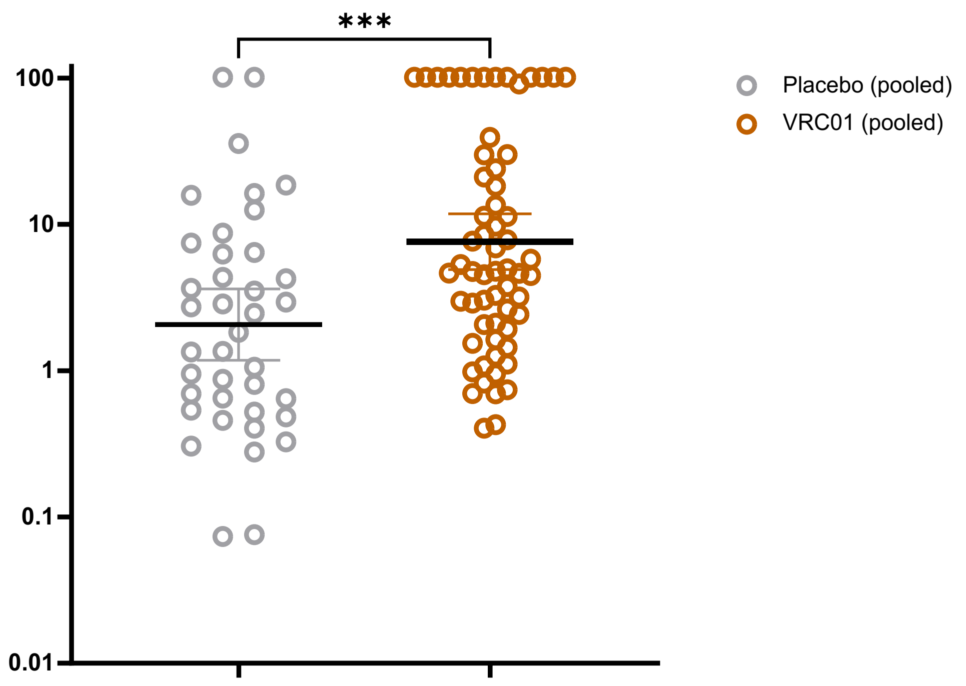

Figure S3. Distribution of IC_{80} for primary endpoint single-lineage cases (trials and treatment groups pooled) single lineage infections. Placebo group shown as grey dots, and the VRC01 group as brown dots. The top and bottom horizontal lines indicated the inter-quartile range, and the bold middle line indicates the median. Median IC_{80} of the placebo group (n=39) was 1.83 µg/ml and the VRC01 group was 4.87 µg/ml (n=62). Columns compared using Mann-Whitney test, two tailed, *** p-value < 0.0005.

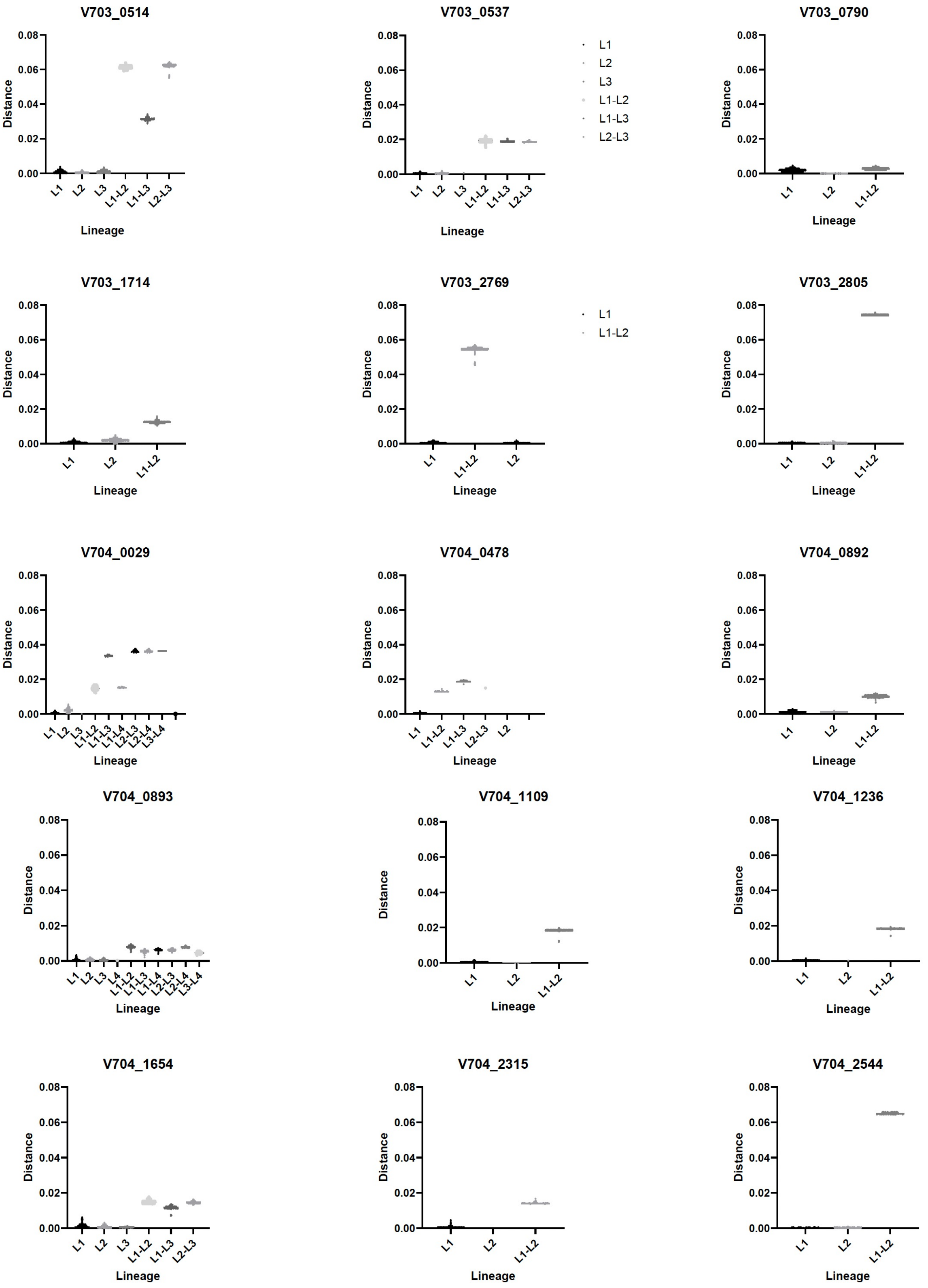

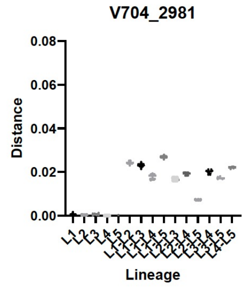

Figure S4. Plots of 16 VRC01 discordant multilineage infections illustrating maximum-likelihood pairwise DNA distances within and between lineages. The medians are indicated by black horizontal lines.

A.

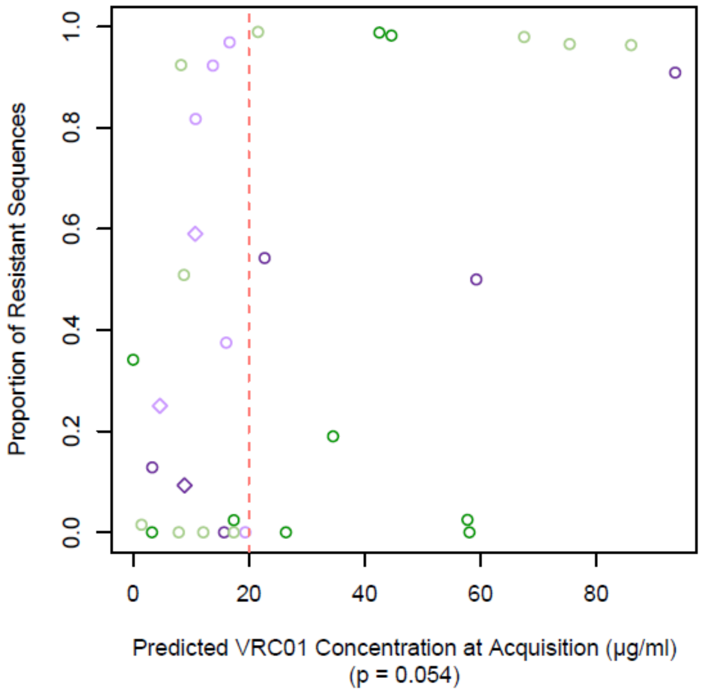

B.

Log10 variance IC80	Trial_Ptid_dose	Sequences assayed (from total lineages)	Sequences from sensitive lineage	Sequences from resistant lineage	Sequences not represented
1.72	V703_0514_10 mg/kg	4 (3)	46 (25%)	108 (59%)	29 (16%)
1.30	V703_2769_10 mg/kg	2 (2)	3 (75%)	1 (25%)	all lineages represented
0.35	V704_1654_30 mg/kg	2 (3)	8 (6%)*	35 (28%	83 (66%)
0.34	V703_1714_30 mg/kg	2 (2)	9 (9%)	85 (88%)	3 (3%)

Ptid = participant id. *IC80 3.1 µg/ml.

Figure S5. A) Proportion of resistant sequences at IC80 > 3. The plot shows the proportion of resistant sequences within each participant (IC80 > 3 as threshold for resistance) on the y-axis against predicted VRC01 concentration at acquisition. **B) Table showing IC80 variance for the four individuals with the greatest IC80 variability (i.e., a larger number of both high and lower values) in participants with a lower predicted VRC01 concentration (< 20 µg/ml).** The table shows the number of lineages assayed (from the total lineages), plus the total number of sequences representing each lineage.

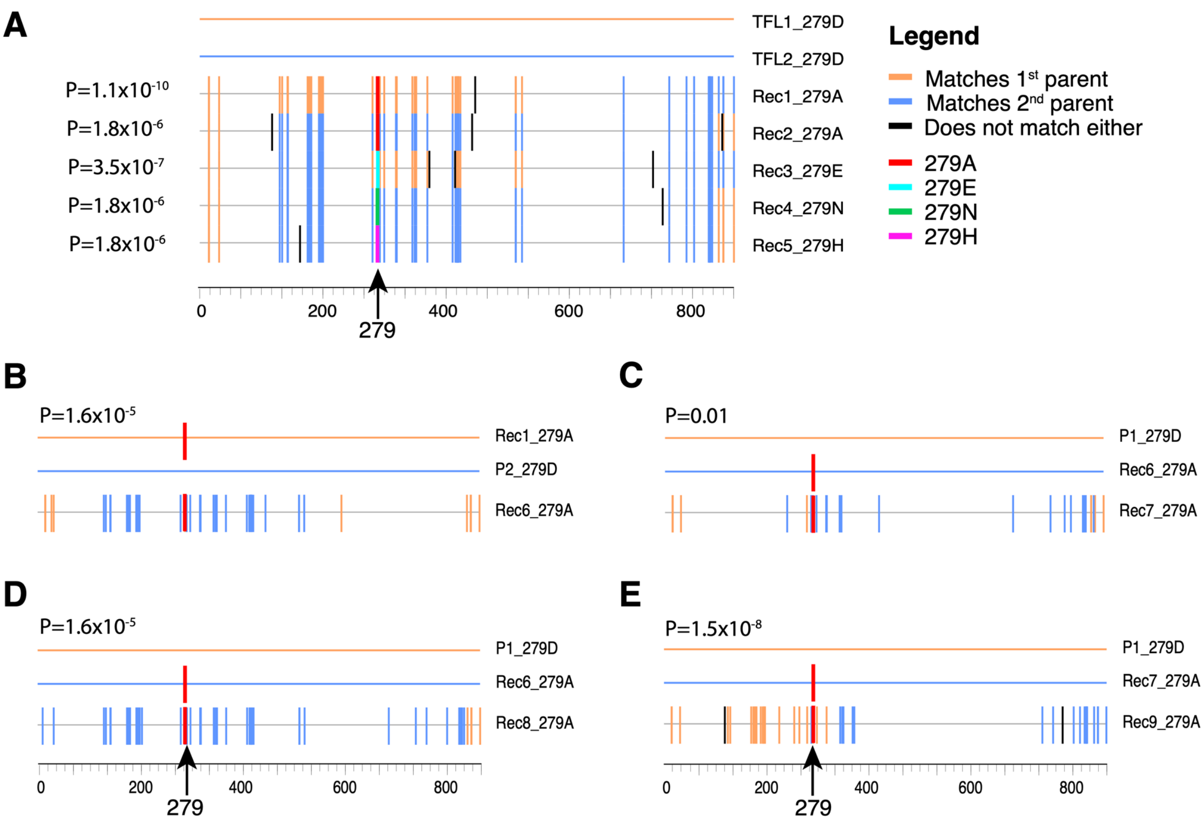

Figure S6: Nine V704_0029 recombinants, each carrying a different escape mutation (D279 A/E/H or N mutation) . Each panel shows recombinant sequence(s) with the two parental strains at the top of each graph and the mutations colored blue or orange according to the parental strain they matched. (A) Five recombinant sequences showing color-coded matching portions of the env gene compared to the two shared parental strains, namely, the most common sequence in the first founder lineage (TFL1, orange) and the most common sequence found in the second founder lineage (TFL2, blue). Both parental lineages carried the 279D amino acid, hence each of the 5 recombinants acquired the escape mutation *de novo* or inherited from a prior parental strain that acquired it following HIV acquisition. Escape mutations are color-coded to illustrate the different amino acid escape variants. (B-E) Four additional recombinant sequence variants in which parental strains carried discordant genotypes at position 459, one sensitive and one resistance mutation, and all the recombinant sequences inherited the resistance mutation. Parental strains are distinct from the founder lineage strains in panel A and indicated as P1 and P2. The second parental lineage is a recombinant itself, and hence these are all second-generation recombination events. P-values on the left of the graph are from the Wald-Wolfowitz Runs test.
