# Supplementary material for: Influence of the broadly neutralizing antibody VRC01 on HIV breakthrough virus populations in antibody-mediated prevention trials
Source: Nat Commun. 2026 Apr 3;17:4780. doi: 10.1038/s41467-026-70888-0 (PMC13219488; doi:10.1038/s41467-026-70888-0)
Supplement: Supplementary file 3 — Reporting Summary [file 41467_2026_70888_MOESM3_ESM.pdf]

Corresponding author(s): Carolyn Williamson (carolyn.williamson@uct.ac.za) James I. Mullins (jmullins@uw.edu)

Last updated by author(s): Feb 20, 2026

Reporting Summary

Nature Portfolio wishes to improve the reproducibility of the work that we publish. This form provides structure for consistency and transparency in reporting. For further information on Nature Portfolio policies, see our Editorial Policies and the Editorial Policy Checklist.

Statistics

For all statistical analyses, confirm that the following items are present in the figure legend, table legend, main text, or Methods section.

n/a	Confirmed	
☐	☒	The exact sample size (*n*) for each experimental group/condition, given as a discrete number and unit of measurement
☐	☒	A statement on whether measurements were taken from distinct samples or whether the same sample was measured repeatedly
☐	☒	The statistical test(s) used AND whether they are one- or two-sided<br>*Only common tests should be described solely by name; describe more complex techniques in the Methods section.*
☐	☒	A description of all covariates tested
☐	☒	A description of any assumptions or corrections, such as tests of normality and adjustment for multiple comparisons
☐	☒	A full description of the statistical parameters including central tendency (e.g. means) or other basic estimates (e.g. regression coefficient) AND variation (e.g. standard deviation) or associated estimates of uncertainty (e.g. confidence intervals)
☐	☒	For null hypothesis testing, the test statistic (e.g. *F*, *t*, *r*) with confidence intervals, effect sizes, degrees of freedom and *P* value noted<br>*Give P values as exact values whenever suitable.*
☒	☐	For Bayesian analysis, information on the choice of priors and Markov chain Monte Carlo settings
☒	☐	For hierarchical and complex designs, identification of the appropriate level for tests and full reporting of outcomes
☐	☒	Estimates of effect sizes (e.g. Cohen's *d*, Pearson's *r*), indicating how they were calculated

Our web collection on statistics for biologists contains articles on many of the points above.

Software and code

Policy information about availability of computer code

Data collection	No software was used
Data analysis	R v4.5 and GraphPad Prism 10

For manuscripts utilizing custom algorithms or software that are central to the research but not yet described in published literature, software must be made available to editors and reviewers. We strongly encourage code deposition in a community repository (e.g. GitHub). See the Nature Portfolio guidelines for submitting code & software for further information.

Data

Policy information about availability of data

All manuscripts must include a data availability statement. This statement should provide the following information, where applicable:

- Accession codes, unique identifiers, or web links for publicly available datasets
- A description of any restrictions on data availability
- For clinical datasets or third party data, please ensure that the statement adheres to our policy

The neutralization and other data underlying the findings of this manuscript are publicly available at the public-facing HVTN data repository at Harvard Dataverse (https://doi.org/10.7910/DVN/3VG3JO), in the files “v703_survival_wk80_tau_sieve.tab” and “v704_survival_wk80_tau_sieve.tab”. The data dictionary for these files is “README_survival.txt”. All individual participant data have been deidentified. All final GP and REN sequences will be deposited in GenBank with Accession numbers (PX890939-PX026359; ON890939-ON891092; PX184492-PX150096 ON980967- ON980814; OR508960- OR508936; OQ912897-OQ912888.1). The GenBank accession numbers for the HIV Env clones used in the TZM-bl target cell neutralization assay are: HVTN 703/HPTN 081 sequences, ON890939–ON891092;

HVTN 704/HPTN 085 sequences, ON980814–ON980967.

Research involving human participants, their data, or biological material

Policy information about studies with human participants or human data. See also policy information about sex, gender (identity/presentation), and sexual orientation and race, ethnicity and racism.

Reporting on sex and gender	N/A
Reporting on race, ethnicity, or other socially relevant groupings	N/A
Population characteristics	N/A
Recruitment	N/A
Ethics oversight	All work described here complied with all relevant ethical regulations. The Institutional Review Boards/Ethic Committees of participating clinical research sites (CRS) approved the studies, which were conducted under the oversight of the NIAID Data Safety Monitoring Board (1). All participants gave written informed consent. The antibody neutralization assays were approved by the Duke University Health System Institutional Review Board (Duke University) through protocol no. Pro00093087 and the University of the Witwatersrand Human Research Ethics Committee (protocol no. M201105). Viral genome sequencing at the University of Cape Town was approved by the UCT Human Research Ethics Committee (HREC reference no. 176/2017) and was considered exempt at the University of Washington.

Note that full information on the approval of the study protocol must also be provided in the manuscript.

Field-specific reporting

Please select the one below that is the best fit for your research. If you are not sure, read the appropriate sections before making your selection.

☒ Life sciences ☐ Behavioural & social sciences ☐ Ecological, evolutionary & environmental sciences

For a reference copy of the document with all sections, see nature.com/documents/nr-reporting-summary-flat.pdf

Life sciences study design

All studies must disclose on these points even when the disclosure is negative.

Sample size	n=172. All primary endpoints included which had HIV sequencing data. Total endpoints for the Africa trial were 74 and two were excluded. Total endpoints for the Americas trial was 98 and all were included.
Data exclusions	two samples were excluded from the Africa trial as there was no sequencing data available.
Replication	Neutralization data was generated as part of the clinical trial, and is published by Corey et al. NEJM, 2021. Data was generated using the TZM-bl assay that has been formally optimized and validated. including equivalency between the NICD and Duke laboratory. To assay for VRC01 sensitivitiy, pre-titrated dose of virus was incubated with serial 3-fold dilutions of the VRC01 drug product in duplicate. This method is described in detail by Corey et al, NEJM 2021.
Randomization	N/A
Blinding	N/A

Reporting for specific materials, systems and methods

We require information from authors about some types of materials, experimental systems and methods used in many studies. Here, indicate whether each material, system or method listed is relevant to your study. If you are not sure if a list item applies to your research, read the appropriate section before selecting a response.

Materials & experimental systems

n/a	Involved in the study	
☒	☐	Antibodies
☐	☒	Eukaryotic cell lines
☒	☐	Palaeontology and archaeology
☒	☐	Animals and other organisms
☐	☒	Clinical data
☒	☐	Dual use research of concern
☒	☐	Plants

Methods

n/a	Involved in the study	
☒	☐	ChIP-seq
☒	☐	Flow cytometry
☒	☐	MRI-based neuroimaging

Eukaryotic cell lines

Policy information about cell lines and Sex and Gender in Research

Cell line source(s)	NA
Authentication	NA
Mycoplasma contamination	NA
Commonly misidentified lines (See ICLAC register)	NA

Clinical data

Policy information about clinical studies

All manuscripts should comply with the ICMJE guidelines for publication of clinical research and a completed CONSORT checklist must be included with all submissions.

Clinical trial registration	ClinicalTrials.gov numbers, NCT02716675 and NCT02568215
Study protocol	PMID: 33730454
Data collection	*Describe the settings and locales of data collection, noting the time periods of recruitment and data collection.*
Outcomes	*Describe how you pre-defined primary and secondary outcome measures and how you assessed these measures.*

Plants

Seed stocks	N/A
Novel plant genotypes	N/A
Authentication	N/A
